# Impact of Domestic Care Environment on Trauma and Posttraumatic Stress Disorder among Orphans in Western Kenya

**DOI:** 10.1371/journal.pone.0089937

**Published:** 2014-03-13

**Authors:** Lukoye Atwoli, David Ayuku, Joseph Hogan, Julius Koech, Rachel Christine Vreeman, Samuel Ayaya, Paula Braitstein

**Affiliations:** 1 Department of Mental Health, School of Medicine, Moi University College of Health Sciences, Eldoret, Kenya; 2 Department of Behavioural Sciences, School of Medicine, Moi University College of Health Sciences, Eldoret, Kenya; 3 Department of Biostatistics, Brown University School of Public Health, Providence, Rhode Island, United States of America; 4 USAID- Academic Model Providing Access To Healthcare (AMPATH) Consortium, Eldoret, Kenya; 5 Department of Children's Health Services Research, Indiana University School of Medicine, Indianapolis, Indiana, United States of America; 6 Department of Child Health and Pediatrics, School of Medicine, Moi University College of Health Sciences, Eldoret, Kenya; 7 Department of Medicine, Indiana University School of Medicine, Indianapolis, Indiana, United States of America; 8 Department of Medicine, School of Medicine, Moi University College of Health Sciences, Eldoret, Kenya; 9 University of Toronto Dalla Lana School of Public Health, Toronto, Ontario, Canada; 10 Regenstrief Institute, Inc., Indianapolis, Indiana, United States of America; University of Toledo, United States of America

## Abstract

**Objective:**

The aim of this study was to determine the impact of the domestic care environment on the prevalence of potentially traumatic events (PTEs) and posttraumatic stress disorder (PTSD) among orphaned and separated children in Uasin Gishu County, western Kenya.

**Methods:**

A total of 1565 (55.5% male) orphaned and separated adolescents aged 10–18 years (mean 13.8 years, sd 2.2), were assessed for PTSD and PTEs including bullying, physical abuse and sexual abuse. In this sample, 746 lived in extended family households, 746 in Charitable Children's Institutions (CCIs), and 73 on the street. Posttraumatic stress symptom (PTSS) scores and PTSD were assessed using the Child PTSD Checklist.

**Results:**

Bullying was the commonest PTE in all domestic care environments, followed by physical and sexual abuse. All PTEs were commonest among the street youth followed by CCIs. However, sexual abuse was more prevalent in households than in CCIs. Prevalence of PTSD was highest among street youth (28.8%), then households (15.0%) and CCIs (11.5%). PTSS scores were also highest among street youth, followed by CCIs and households. Bullying was associated with higher PTSS scores and PTSD odds than either sexual or physical abuse.

**Conclusion:**

This study demonstrated differences in distribution of trauma and PTSD among orphaned and separated children in different domestic care environments, with street youth suffering more than those in CCIs or households. Interventions are needed to address bullying and sexual abuse, especially in extended family households. Street youth, a heretofore neglected population, are urgently in need of dedicated mental health services and support.

## Introduction

The burden of orphanhood is high and continues to rise in most low and middle income countries, fuelled to a significant extent by the HIV/AIDS pandemic and adverse socio-political circumstances [1], [2]. In 2010, the United Nations Children's Fund estimated that Africa was home to over 53 million orphans from all causes, with HIV/AIDS being responsible for nearly 30% of these [2]. The number of orphans in Kenya was estimated at 2.5 million in 2010, and little documented action was being taken to assess and address problems affecting their well-being [2].

Kenya's recent history of political violence has also resulted in an increased number of orphaned, displaced and separated children. Despite early interventions to address immediate psychological and social needs in Eldoret at the time [3], many displaced children remained in camps for long periods of time, increasing the risk of trauma and PTSD. A recent study among impoverished youth affected by postelection violence in Kenya reported prevalence rates of PTSD of 18% using a screening tool, and 12% using a clinical interview [4]. Another study found that over 90% of rural Kenyan youth had been exposed to a potentially traumatic event, and 34.5% of the total sample met criteria for PTSD [5].

Posttraumatic stress disorder (PTSD) is common among orphaned and separated children. A series of studies from South Africa showed a high prevalence of significant posttraumatic stress symptoms among children orphaned by HIV/AIDS, with estimated PTSD rates as high as 30–73% [6], [7]. In Rwanda, ten years after the 1994 genocide, the prevalence rate of PTSD among children orphaned during the genocide and living either in child-headed households or in orphanages was 44% [8]. Similarly, a study among war-traumatised children in Bosnia and Herzegovina found a PTSD prevalence rate of 51.6% [9].

A number of studies have examined the impact of the domestic care environment on the presence of these symptoms. In the Rwandan study, orphans living in child-headed households were shown to have the greatest vulnerability for developing PTSD [8]. A study from Iraqi Kurdistan, comparing orphanage care with traditional foster care environments, found a higher frequency of PTSD among children in orphanages than in foster care [10]. In Bosnia and Herzegovina, PTSD rates were highest among orphans living with the surviving parent, followed by those who had lost a relative and were living in an NGO-sponsored children's village [9]. Losing both parents was associated with the greatest risk of developing PTSD.

Very few studies comparing the exposure to PTEs and occurrence of PTSD among orphans and separated children in diverse living environments have been carried out in low and middle income countries and outside of conflict situations. The present study therefore aimed to determine the prevalence and severity of PTEs and posttraumatic stress symptoms among orphaned and separated children in a non-conflict setting, and the impact of the care environment on these outcomes.

## Methods

### Ethics statement

This study was approved by the Moi University College of Health Sciences and the Moi Teaching and Referral Hospital Institutional Research and Ethics Committee, as well as the Indiana University Institutional Review Board. Depending on the care environment, informed consent was provided by the head of household, Director of CCI, and in the case of the street youth, by the District Children's Officer (DCO). Individual written assent was provided by all the children in this study. Fingerprint impressions were used for both children and guardians who were unable to sign or write their name. The study dataset contained no identifying information, and all research data was kept confidential. This consent procedure was approved by the Moi University College of Health Sciences and Moi Teaching and Referral Hospital Institutional Research and Ethics Committee, as well as the Indiana University Institutional Review Board.

### Study setting

The Orphaned and Separated Children's Assessments Related to their (OSCAR's) Health and Well-Being Project (1R01HD060478-01A1) is a 5-year longitudinal cohort study evaluating the effects of different care environments on the physical and mental health outcomes of orphaned and separated children aged 18 years of age or less. The detailed study methodology has been published elsewhere, and only a brief description is provided here [11].

The study catchment area is the Uasin Gishu (UG) County, one of the 47 counties of Kenya, located approximately 300KM to the Northwest of Kenya's capital city, Nairobi. In 2010, UG County had approximately 894,179 individuals from 202,291 households, of whom 41.5% are aged 14 years or less [12]. The city of Eldoret is the County's capital, administrative and commercial centre. Eldoret has a total population of 289,389 and is currently, the 5th largest city in the country.

The OSCAR study describes the different domestic care environments for orphans and separated children, determining whether they are able to meet basic socioeconomic needs of the resident children, and examining the effect of care environment on resident children's physical and mental health over time. The study began enrolling participants in July 2010, and the current analysis is based on baseline data collected between July 2010 and June 2012.

### Study population

The project follows a cohort of orphaned and separated children from communities within 8 locations, 300 households, 19 Charitable Children's Institutions (CCIs), and 100 street-involved children and youth in UG County. Psychosocial assessments (including PTSD assessment) were done for participants who were aged 10 and 18 years, able to communicate in English or Kiswahili (the languages in which the assessments were carried out) and to give consent for the assessment to be carried out. Those with current severe symptoms of mental or general medical conditions were excluded from the assessments and instead offered treatment at the referral hospital.

Households taking care of orphans or separated children were recruited using stratified random sampling preceded by extensive community consultations [11] with the aid of Community Health Workers. As described in a previous publication [11], community consultations involved use of community meetings (known as mabaraza in Kiswahili) as well as smaller discussions with community leaders and a community advisory board. Consenting, registration, enrolment and all individual study procedures for recruited households took place at the central OSCAR clinic located at the Moi Teaching and Referral Hospital (MTRH) in Eldoret, Kenya. All children in the households were included in the study in order to minimise any distress or stigma associated with selection to participate in the study.

Under the Kenyan Children Act (2001), orphanages and other institutions serving orphans are called CCIs (i.e. charitable children's institutions) if they are able to accommodate ≥20 or more children [13]. All such institutions in UG County subject to the Children Act (2001) were eligible for participation in and recruitment in the study. The UG County Children's Department maintains a list of registered and unregistered institutions, and holds monthly meetings with them in the UG Children's Services Forum. In total, there were 21 CCIs identified in the UG County that chose to participate in the study, including one foster home operated by the Directors of an official CCI and a community-based organization supporting orphaned and vulnerable youth. One CCI declined to participate. All children, including the biological offspring of CCI personnel (e.g. children of so-called House Parents) were eligible to participate. All study procedures for the children in CCIs took place at the respective institutions.

Street youth were recruited directly from the street, as well as through community-based organizations using snowball methods by a trained and experienced street youth outreach worker. Children and youth aged ≤18 years of age willing to participate were referred to the central OSCAR clinic for assenting and registration. All study procedures for the street youth took place at the central clinic.

After complete description of the study to the subjects, written informed consent was obtained as described below.

### Data collection

Socio-demographic data were collected by a study social worker using a standardized form designed by the researchers to collect information about the age, gender, living circumstances, orphan status, current caregiver and length of time with current caregiver, as well whether a parent died due to HIV/AIDS. The socio-demographic characteristics are presented in **Table 1**.

**Table 1:** Socio-demographic characteristics N = 1565.

	Whole Population	CCI, n = 746	HH, n = 746	SC, n = 73		
	Mean (SD)	Mean (SD)	Mean (SD)	Mean (SD)	P-value	Partial Eta-squared
**Age**	13.8 (2.2)	13.7 (2.2)	13.8 (2.2)	14.3 (2.1)	0.116	0.003
**Other Variables**	**n (%)**	**n (%)**	**n (%)**	**n (%)**		**Phi Coefficient**
**Sex**						
Male	869 (55.5)	426 (57.1)	383 (51.3)	60 (82.2)		
Female	696 (44.5)	320 (42.9)	363 (48.7)	13 (17.8)	**<0.0001**	0.13
**Orphan status**						
Single orphan or separated	853 (54.5)	341 (45.1)	476 (63.8)	36 (49.3)		
Double orphan and separated	601 (38.4)	392 (52.6)	174 (23.3)	35 (48.0)		
Both parents alive & child is living with them	105 (6.7)	8 (1.1)	95 (12.7)	2 (2.7)		
Missing	6 (0.4)	5 (0.7)	1 (0.1)	0 (0.0)	**<0.0001**	0.35
**Length of time with current caregiver**						
<6 months	103 (6.7)	85 (11.4)	9 (1.2)	9 (12.3)		
6months-2years	185 (11.8)	153 (20.5)	14 (1.9)	18 (24.7)		
2–5 years	342 (21.9)	237 (31.8)	81 (10.9)	24 (32.9)		
>5 years	905 (57.8)	260 (34.9)	633 (84.9)	12 (16.4)		
Missing	30 (1.9)	11 (1.5)	9 (1.2)	10 (13.7)	**<0.0001**	0.48
**HIV status**						
Positive	33 (2.1)	20 (2.7)	13 (1.7)	0 (0.0)		
Negative	1495 (95.5)	708 (94.9)	717 (96.1)	70 (95.9)		
Unknown/indeterminate	28 (1.8)	16 (2.1)	9 (1.2)	3 (4.1)		
Missing	9 (0.6)	2 (0.3)	7 (0.9)	0 (0.0)	**<0.0001**	0.08

Potentially traumatic events (PTEs) were assessed using items in the International Society of the Prevention of Child Abuse and Neglect (ISPCAN) Child Abuse Screening Tool for Children (ICAST-C), which assesses different types of violence against children [14]. The ICAST-C was developed by ISPCAN (http://www.ispcan.org/) in partnership with UNICEF and the United Nations Secretary General's Study on violence against children. In the present study, we selected items assessing sexual abuse (being made to look at another person's private parts or having another person look at the child's, being touched in their private parts or being made to touch another person's, being forced to have sex) and physical abuse (being threatened with injury, being hit hard enough to cause injury, being severely injured by scalding, burning or attempted drowning). Questions about bullying were extracted from an instrument used by Cluver et al [15] among South African children, and included in this study as PTEs.

Posttraumatic stress symptom (PTSS) score was calculated based on the Child PTSD Checklist [16], a 28-item scale based on DSM IV TR criteria. Each item is scored either 0 (Never), 1 (Sometimes), 2 (Often) or 3 (All the time). The total score ranges from 0 to 84, with higher scores indicating greater symptom occurrence and severity.

Posttraumatic stress disorder (PTSD) was diagnosed based on the DSM IV TR criteria, using the Child PTSD Checklist items as the symptom indicators. Since the Child PTSD Checklist does not have a trauma checklist (Criterion A1) and items related to response (Criterion A2), the duration of symptoms (Criterion E) and distress or impairment (Criterion F), it is not possible to use this checklist to make a clinical diagnosis of PTSD. However, a child meeting criteria B, C and D has a high likelihood of having PTSD, and it has been demonstrated in South African studies that the checklist is a useful screening tool for the condition [7], [17]. Additionally, to approximate criterion A1 of the DSM IV TR criteria, PTSD was assessed in relation to the PTEs discussed above, including sexual abuse, physical abuse and bullying items. Respondents reporting lifetime occurrence of any of these PTEs were asked about Criterion B, C and D symptoms occurring in the past one month. In this study, as in the previous studies using the same instrument, we used a cut-off of one re-experiencing symptom, three avoidance or numbing symptoms, and two hyperarousal symptoms. Each item on the checklist had to meet the conservative symptom threshold of ‘most of the time’ (a score of 2 or more) to be considered to have met the criterion.

All trauma and PTSD interviews were conducted by staff trained in mental health assessments with a background in psychiatry, clinical psychology and counselling.

### Data analysis

Out of the 1565 participants enrolled in the study, analyses were conducted only for the 1451 (92.7%) records that had complete data for all variables of interest, as shown in **Table 2**. The 114 cases with missing data were randomly distributed and did not differ significantly from those with complete data.

**Table 2:** PTEs, PTSD and PTSS median score variation with sex, stratified by care environment, N = 1451^a^.

	Households					CCI					Street children				
	ALL (n = 681)	Male (n = 352)	Female (n = 329)			ALL (n = 704)	Male (n = 401)	Female (n = 303)			ALL (n = 66)	Male (n = 55)	Female (n = 11)		
PTEs	n (%)	n (%)	n (%)	P-Value	Phi Coef	n (%)	n (%)	n (%)	P-Value	Phi Coef	n (%)	n (%)	n (%)	P-Value	Phi Coef
Bullying	**511 (75.0)**	268 (76.1)	243 (73.9)	0.493	0.03	**588 (83.5)**	337 (84.0)	251 (82.8)	0.670	0.02	**58 (87.9)**	49 (89.1)	9 (81.8)	0.500	0.08
Physical abuse	**176 (25.8)**	107 (30.4)	69 (21.0)	**0.005** *	0.11	**229 (32.5)**	152 (37.9)	77 (25.4)	**0.001** *	0.13	**43 (65.2)**	36 (65.5)	7 (63.6)	0.908	0.01
Sexual abuse	**119 (17.5)**	69 (19.6)	50 (15.2)	0.130	0.06	**87 (12.4)**	52 (13.0)	35 (11.6)	0.572	0.02	**23 (34.9)**	18 (32.7)	5 (45.5)	0.419	0.10
**PTSD prevalence**	**102 (15.0)**	58 (16.5)	44 (13.4)	0.257	0.04	**81 (11.5)**	50 (12.5)	31 (10.2)	0.357	0.03	**19 (28.8)**	15 (27.3)	4 (36.4)	0.543	0.07
**Median (IQR)**	**Median (IQR)**	**Median (IQR)**	**Median (IQR)**			**Median (IQR)**	**Median (IQR)**	**Median (IQR)**			**Median (IQR)**	**Median (IQR)**	**Median (IQR)**		
**PTSS Score**	**12 (2–23)**	11 (1–23)	12 (3–23)	0.855		**14 (7–23)**	14 (7–23)	14 (6–23)	0.890		**16 (7–26)**	16 (6–26)	21 (10–32)	0.496	

a 114 cases with incomplete data excluded; Overall, PTEs, PTSD prevalence rates and PTSS median scores varied significantly across the care environments (p<0.01).

*Statistically Significant, p<0.01.

The primary outcomes for this analysis were Posttraumatic stress symptom (PTSS) score and Posttraumatic stress disorder (PTSD) respectively. PTSS score was calculated by summing each item on the Child PTSD Checklist, which has 28 items each with a score ranging from 0 to 3. PTSD was evaluated using the DSM IV TR criteria as explained under the data collection section. Parametric and non-parametric descriptive statistics were employed to summarize both categorical and continuous variables. For continuous variables, mean and median together with standard deviation and inter-quartile range were calculated, respectively. The Chi-Square test was used to test for associations between categorical or dichotomous variables. When the expected frequencies were lower than 5 for the chi-square tests, the results were controlled with Fisher's Exact-Test. T-tests and analysis of variance (ANOVA) were also used to compare means across the groups (gender and types of care environments).

To assess risk factors associated with PTSS score and PTSD, both median and logistic regression models were employed.

To assess the impact of the care environment on the PTSS score, two median regression models were calculated (**Table 3**). In the first model, we performed unadjusted median regression with the type of care environment as a predictor. In the second model, the effect of care environment was assessed while controlling for age and gender. Similarly, to assess the impact of care environment on the prevalence of PTSD, an unadjusted logistic regression model was calculated for presence of PTSD with care environment as a predictor, followed by a second model adjusted for age and gender.

**Table 3:** PTSS Median Score and PTSD association with care environment.

	PTSS Score Median Regression Coefficient		PTSD Odds Ratio	
Covariates	Unadjusted coefficient (95%CI)	Adjusted coefficient (95%CI)	Unadjusted OR (95%CI)	Adjusted OR (95%CI)
**HH** a	Reference	Reference	Reference	Reference
**CCI**	2.00 (−0.06, 4.06)	**2.49 (0.40, 4.57)** *	0.74 (0.54, 1.00)	0.73 (0.48, 1.09)
**SC**	**5.00 (0.08, 9.92)** *	3.97 (−1.03, 8.97)	**2.25 (1.27, 3.98)** *	**2.14 (1.20, 3.82)** *
Age	–	1.11 (0.64, 1.58)	–	0.96 (0.90, 1.02)
Sex	–	−0.43 (−2.49, 1.64)	–	1.26 (0.94, 1.66)

a Reference category.

*Statistically Significant, p<0.01.

Further models were calculated for the impact of specific PTEs on PTSS median scores and on the prevalence of PTSD. All these models were adjusted for age, gender and care environment, and are presented in **Table 4**.

**Table 4:** Independent models adjusted for age, sex and care environment examining variation of PTSS median coefficient and PTSD with different PTEs.

	Bullying	Physical Abuse	Sexual Abuse	Bullying	Physical Abuse	Sexual Abuse
	Adjusted Coefficient (95%CI)	Adjusted Coefficient (95%CI)	Adjusted Coefficient (95%CI)	Adjusted OR (95%CI)	Adjusted OR (95%CI)	Adjusted OR (95%CI)
**Coefficient/OR**	**11.04 (9.23, 12.86)** *	**8.94 (7.17, 10.70)** *	**9.63 (7.10, 12.15)** *	**7.47 (3.63, 15.37)** *	**3.01 (2.15, 4.20)** *	**3.47 (2.14, 5.62)** *
**Covariates**						
Age	1.01 (0.67, 1.34)	−1.17 (0.80, 1.53)	1.09 (0.67, 1.51)	0.95 (0.89, 1.01)	0.96 (0.90, 1.03)	0.96 (0.90, 1.02)
Sex	−0.99 (−2.46, 0.49)	−1.21 (−3.31, 0.89)	−0.34 (−2.20, 1.53)	1.22 (0.91, 1.63)	1.11 (0.82, 1.48)	1.21 (0.90, 1.63)
**Care Environment**						
HH^a^	Reference	Reference	Reference	Reference	Reference	Reference
CCI	0.82 (−0.68, 2.31)	1.63 (−0.01, 3.27)	2.84 (0.96, 4.73)	0.64 (0.43, 0.96)	0.66 (0.45, 0.97)	0.79 (0.54, 1.15)
SC	3.31 (−0.25, 6.87)	2.14 (−1.85, 6.13)	2.38 (−2.14, 6.90)	1.86 (1.04, 3.33)	1.51 (0.83, 2.78)	1.84 (0.98, 3.45)

*Statistically Significant, p<0.01.

a Reference category.

## Results

### Socio-demographic characteristics

A total of 1565 participants were recruited into the study at baseline, including 746 from households, 746 from CCIs, and 73 street children and youth. The mean age was 13.8 years, and 55.5% of the participants were male. The majority of participants (54.5%) were single orphans, and most had lived over five years with the present caregiver (57.8%). Over 95% of the children were HIV-negative.

As shown in **Table 1**, children in the three care environments did not differ significantly in age, but there were variations in sex, orphan status, length of time with current caregiver and HIV status.

A total of 114 (7.3%) cases had incomplete data on any one of the socio-demographic and trauma variables, and are not included in the following analyses.

### Potentially traumatic events

In this study, we examined three potentially traumatic event (PTE) classes among the participants: bullying, physical abuse and sexual abuse.

As described in **Table 2**, bullying was the most common PTE across all care environments (87.9% among street youth, 83.5% among those in CCIs and 75.0% among those in households). Street youth had the highest prevalence rates of all the PTEs, while adolescents in households reported significantly higher rates of sexual abuse than those in CCIs. Among adolescents in households and in CCIs, boys reported significantly higher rates of physical abuse than girls. There was no statistically significant variation in the occurrence of the other PTEs with gender.

### PTSD and PTSS scores

As shown in **Table 2**, street youth had a statistically significantly higher prevalence of PTSD (28.8%, p<0.05), than those in households (15.0%) or CCIs (11.5%). They also had significantly higher PTSS median scores (16.0, IQR 7.0–26.0) compared to those in households (12.0, IQR 2.0–23.0) or CCIs (14.0, IQR 7.0–23.0).

**Table 3** shows that controlling for age and sex, street youth were more than twice as likely as those living in households to have PTSD (AOR 2.14, CI 1.20–3.82). Children in CCIs had median PTSS scores about 2.5 points higher than those in households (p<0.005). Although street youth also had elevated scores compared to those in households, the effect was not statistically significant likely due to the relatively small number of street youth participants.

**Table 4** shows the results of six independent median and logistic regression models examining the variation of PTSS median score and PTSD prevalence with exposure to different PTEs. Adjusting for age, sex and care environment, bullying resulted in the greatest rise in the PTSS scores (11.04, CI 9.23–12.86) compared to physical abuse (8.94, CI 7.17–10.70) or sexual abuse (9.63, CI 7.10–12.15).

In relation to PTSD diagnosis, bullying increased the odds ratio of PTSD by a factor of about 7.5 compared with those not reporting bullying (AOR 7.47, CI 3.683–6.429). Physical abuse was associated with a roughly threefold rise in the odds of PTSD (3.28, CI 2.646–4.058, p<0.001), as was sexual abuse (2.82, CI 2.115–3.761, p<0.001).

## Discusssion

This study has established that a quarter of the street youth participating in this study screened positive for PTSD, and are more than twice as likely as orphaned children living in extended family households to have PTSD. Orphaned children living in institutional environments in this setting are no more likely than orphaned children living in extended family households to have PTSD.

Another critical finding of this study is the high prevalence of bullying and its strong association with PTSD across all care environments, including in household settings where many, including the victims, tend to minimize its impact [18]. Three quarters of children living with relatives in households reported having been bullied. Adolescents living in CCIs, and street youth, had similar rates of bullying, both over 80%. Bullying in households (by siblings) has been shown elsewhere to be just as significant as peer aggression in other settings in causing distress in children and adolescents [19]. In the present study, bullying increased the odds ratios for PTSD over seven times, while raising the PTSS score by over 11 points compared to children who were not bullied. Future research on children and adolescents must evaluate bullying at home and in other settings as a potentially traumatic event due to its high prevalence and the significant association with PTSD. In addition, effective interventions to address bullying in this setting are needed.

Not surprisingly, street youth had significantly higher rates of all PTEs and PTSD, as well as higher PTSS scores compared to adolescents in other care environments. Indeed, previous studies have found a high prevalence of both trauma and PTSD among the homeless population [20], [21]. However, the direction of the association has been more difficult to determine. North et al [20] reported that childhood histories of abuse and family fighting were predictive of both traumatic events and PTSD, and suggested that factors leading to PTSD began long before the onset of homelessness and might overlap with factors operative in the genesis of homelessness. Since the OSCAR study is longitudinal, we may be able to provide more insights in this area at the conclusion of the five-year follow-up period.

In UG County, most children who enter CCIs are primarily from homes suffering extreme adversity, including death of primary caregivers, and child abuse and neglect [22]. It is therefore not surprising that children in CCIs had a higher prevalence of bullying and physical abuse compared to those in households. However, the prevalence rate of PTSD was significantly higher among adolescents in households than those in CCIs, perhaps reflecting the impact of the types of abuse common in households. Instructively, children in households had higher rates of sexual abuse than those in CCIs, a finding that is quite in tandem with previous reports that AIDS-orphanhood predicts, among other outcomes, transactional sexual exploitation especially in poor households [23]. The suggestion that orphaned children living with relatives are more vulnerable to sexual abuse than those in CCIs calls for further research to elucidate the nature of this sexual abuse, and to develop appropriate interventions to reduce this risk.

Children in CCIs also had PTSS scores 2.5 points higher than those in households, although there was no statistically significant difference in the odds of PTSD between the two care environments. This suggests that they had more severe symptoms compared to children in households, a finding that would be in keeping with the fact that many of the children in CCIs are from households that have had severe social and economic challenges. Such factors would also independently predict higher rates of trauma and PTSD [20]. Conversely, although street youth had significantly increased odds of having PTSD, the adjusted PTSS scores were not different from those of adolescents in households. This again suggests that although street youth may have enough symptoms to meet the criteria for PTSD, the symptom severity did not differ significantly from those of their counterparts living with relatives.

This study is among the first to describe the association between different care environments for orphans and separated children and the occurrence of PTEs and PTSD in Africa. Among the strengths in this study are the relatively large sample size of youth in different care environments, inclusion of street youth and the more comprehensive assessment of potentially traumatic events, including bullying.

These findings must necessarily be interpreted with caution, given a number of limitations. Firstly, causal attribution cannot be made from cross-sectional data such as these. The associations we report can only form the basis of future longitudinal analyses to confirm their directionality. Secondly, the findings may not be generalisable to the generation population in Kenya due to key differences between orphans and separated children elsewhere and those in our study. For instance, street children were not randomly sampled due to difficulties in determining their location at any one point during data collection, and therefore do not represent the entire population of street children in UG County, or indeed in the country.

Finally, the study instruments employed in this study were not validated in this study population, and the findings may not accurately reflect the extent of PTSD symptoms in this population. However, the instruments have been validated and used extensively in similar populations in South Africa, reducing the need to actively validate them in our context.

Notwithstanding these limitations, our comparative findings serve as an important starting point in identifying the problems faced by this group of neglected children, and perhaps provide a template for a viable intervention.

## Conclusion

This study has demonstrated very high rates of traumatisation and PTSD among orphans in different domestic care environments, with street youth suffering more than adolescents in CCIs or households. We recommend that new strategies are needed to address sexual abuse and bullying in order to reduce the burden of trauma and PTSD among orphaned and separated adolescents. Our study significantly expands the body of knowledge on the mental health of orphans and separated youth, and the large sample size provides sufficient power to describe interactions between care environments and trauma and PTSD. We strongly recommend further studies to confirm the findings in this study, and hope that longitudinal data may begin to address some of these needs. Street youth, a heretofore neglected population, are urgently in need of dedicated mental health services and support.
